# Cisd1 synergizes with Cisd2 to modulate protein processing by maintaining mitochondrial and ER homeostasis

**DOI:** 10.18632/aging.206249

**Published:** 2025-05-08

**Authors:** Yi-Fan Chen, Yuan-Chi Teng, Jian-Hsin Yang, Cheng-Heng Kao, Ting-Fen Tsai

**Affiliations:** 1 International Master Program for Translation Science, College of Medical Science and Technology, Taipei Medical University, New Taipei City 23564, Taiwan; 2 The Ph.D. Program for Translational Medicine, College of Medical Science and Technology, Taipei Medical University, Taipei 11529, Taiwan; 3 TMU Research Center of Cancer Translational Medicine, Taipei Medical University, Taipei 11031, Taiwan; 4 International Ph.D. Program for Translational Science, College of Medical Science and Technology, Taipei Medical University, Taipei 11031, Taiwan; 5 Master Program in Clinical Genomics and Proteomics, School of Pharmacy, Taipei Medical University, Taipei 11031, Taiwan; 6 Department of Life Sciences and Institute of Genome Sciences, National Yang Ming Chiao Tung University, Taipei 112, Taiwan; 7 Department of Medical Research, Taipei Veterans General Hospital, Taipei 112, Taiwan; 8 Center of General Education, Chang Gung University, Taoyuan 333, Taiwan; 9 Center for Healthy Longevity and Aging Sciences, National Yang Ming Chiao Tung University, Taipei 112, Taiwan; 10 Institute of Molecular and Genomic Medicine, National Health Research Institutes, Zhunan 350, Taiwan

**Keywords:** skeletal muscle, Cisd1, Cisd2, knockout mice, protein process, ER stress, mitochondria

## Abstract

Connection and crosstalk among the organelles critically contribute to cellular functions. Destruction of any kind of organelle is likely to induce a series of intracellular disorders and finally lead to cell death. Because of its subcellular locations, CDGSH iron-sulfur domain-containing protein 1 (Cisd1) and Cisd2 have functions that are related to maintaining mitochondria and ER homeostasis. As previous reports have shown, Cisd2 knockout mice have a decreased body weight and poor survival rate, and the primary defects were conducted in skeletal muscle. Our previous findings indicated that Cisd1 deletion causes a range of skeletal muscle defects in mice with Cisd2 deficiency, including mitochondrial degeneration, endoplasmic reticulum (ER) stress, and alteration of protein process, as well as programmed cell death. In Cisd1 and Cisd2 deficient condition, the whole of the protein biosynthesis was damaged, including translation, modification, transport, and degradation. Changes in the immune response, redox regulation, and metabolism were also present in Cisd1 and Cisd2 double knockout mice. Overall, we have demonstrated that Cisd1 and Cisd2 knockout have a synergistic effect on skeletal muscles, and that Cisd2 plays a more critical role than Cisd1. These synergistic effects impact signaling regulation and interrupt the crosstalk and homeostasis of organelles. This creates severe disorders in various tissues and organs.

## INTRODUCTION

Initiation and regulation of protein translation in eukaryotic cells needs to be well-controlled to avoid nutrient deprivation and stress, development and differentiation, nervous system dysfunction, aging, and disease. Protein synthesis is a molecular process that requires high energy and requires careful regulation, thence this process needs a range of sensors and factors to control the whole process. For example, the phosphorylation of eukaryotic initiation factor 2A (eIF2α) by stress-activated eIF2α kinases is the intracellular sensor of nutrient availability and this is closely linked to the rate of protein synthesis [1]. Post-translational modification (PTM) is an important process that occurs in the membrane and lumen of the endoplasmic reticulum (ER) and includes the formation of disulfide bonds, the proper folding of proteins, the addition and processing of carbohydrates to proteins, various specific proteolytic cleavages, and the assembly of subunits into multimeric proteins. Furthermore, some aspects of the modification processes take place in the Golgi complex. Specific coat proteins help to transport other proteins from the rough ER to the Golgi apparatus [2]. When proteins are not properly folded, they are tagged with ubiquitin and destroyed by proteasomes [3]. Dysfunction of the ubiquitin-proteosomes system (UPS) leads to defects in mitochondrial morphology and functions; relatively aberrantly high levels of reactive oxygen species (ROS) are produced by the mitochondria, and these contribute to an induction of the UPS [4].

Some situations, such as an inhibition of protein processing, promote misfolding and/or damage to proteins and this then triggers a series of stress responses, including an acute inhibition of mRNA translation, the subsequent induction of various protein chaperones, and a recovery of mRNA translation. Stress responsive systems mostly observed in the ER and when ER stress cannot be reversed, this usually leads to cellular dysfunction and cell death [5]. In addition to apoptosis, many forms of non-apoptotic cell death (also known as necrotic cell death), such as necroptosis, pyroptosis, ferroptosis, and NETosis, are programmed and cause a range of immunogenic effects, particularly the induction of proinflammatory responses [6]. These forms of programmed necrosis are mediated by an activatable genetically regulated cell death program, and they have shared morphological features, including plasma membrane permeabilization, cellular swelling, the release of harmful cellular content and the induction of inflammation [7]. Some evidence indicated that non-apoptotic cell death is the backup cell death process when apoptosis is impeded [8].

Cisd1 and Cisd2 are the members of the CDGSH (Cys-Asp-Gly-Ser-His) iron-sulfur (Fe-S) domain-containing protein family [9, 10]. Only three members are currently known in this protein family. Cisd1 has been reported to be localized on mitochondrial outer membrane. In contrast, Cisd2 is not only located on the mitochondrial outer membrane, but on the mitochondria-associated ER membranes (MAMs) and the ER [11, 12]. MAMs connect two independent organelles, the mitochondria and the ER, and contribute to various functions, such as apoptosis, calcium metabolism, cellular homeostasis and systemic energy metabolism [13, 14]. Alterations affecting MAM proteins are highly related to the progression of various metabolic diseases. The content of MAMs contributes to insulin sensitivity and glucose metabolism. Meanwhile the impairment of the ER and mitochondria connection leads to mitochondrial dysfunctions in skeletal muscle. If genetic deletion of a structural protein damages MAM integrity, glucose uptake, lipid metabolism and insulin signaling are disrupted in skeletal muscle [15, 16]. The essential role of MAMs in maintaining material exchanges between the mitochondria and the ER, which affects cellular homeostasis, means that a disruption of MAMs can eventually result in cell death [17].

Our previous studies have shown that Cisd2 deletion in skeletal muscles caused activation of the UPR (unfolded protein response) induces ER stress, affects calcium signaling and alters redox regulation [18]. Cisd2 conventional knockout mice have various severe defects that primarily affect skeletal muscle, cardiac muscle, and the neuronal system [12]; this model is a mouse model that shows premature aging phenotypes which are exhausted with time. Cisd1 deletion in mice has been reported to destroy mitochondrial functions, resulting in decreasing striatum dopamine levels and increasing ROS generation and iron accumulation [19]. Both Cisd1 and CIsd2 have been demonstrated to play a role in iron, calcium, and ROS homeostasis in different model systems [20], and both are involved in iron metabolism, indicating that they are potential regulators of ferroptosis. Cisd1 suppresses lipid peroxidation in mitochondria and protects cells from ferroptosis [21], while Cisd2 is also associated with the resistance to ferroptosis [22]. The genetic ablation of Cisd1 or of Cisd2 leads to an abnormal accumulation of iron together with the presence of lipid peroxidation products in mitochondria [23]. In addition, Cisd1 and Cisd2 possibly have antioxidant functions by being involved in the transfer of iron from mitochondria to other places in the cell, and a reduction of Cisd1 and/or Cisd2 is known to trigger ROS generation because of the presence of an iron overload organelles [24]. It is well known that the Cisd family are evolutionary conserved and consists of three genes (Cisd1, Cisd2 and Cisd3) in mammalians. Two of these genes, Cisd1 and Cisd2, have evolutionally similar structures and may participate in the same or related cellular mechanisms and pathways; specifically, they may have critical functions that affect the mitochondria-MAMs-ER balance. In this study, we expect Cisd1 and Cisd2 to have synergistic effects on the mechanisms and functions of skeletal muscles, and we would like to verify the critical signaling roles using appropriate genetically modified mouse models.

## MATERIALS AND METHODS

### Mice

Cisd1 KO mice were generated by Transgenic mouse models core facility (A4) of MOST and their genotype confirmed by Southern blotting and PCR. The Cisd1 KO mice was generated in R1 ES cells and then backcrossed to C57BL/6 background. Cisd1 was completely deleted in the Cisd1 KO mice. Cisd2 KO mice were generated as previously described [12]. Cisd1 +/− female mouse was mated with Cisd2 +/− male mouse to generate Cisd1 and Cisd2 double knockout (Cisd1&2 DKO) mice. Wild-type (WT) mice in this study were the littermates of the Cisd1&2 DKO mice. All mouse lines have congenic C57BL/6 background and were maintained in a specific pathogen–free facility with a 12/12 h light/dark cycle at 20°C to 22°C at the Laboratory Animal Center, National Yang Ming Chiao Tung University. Mice were weighed weekly starting at the age of 2 weeks. At the time of sampling, the mice were euthanized by carbon dioxide inhalation. The animal protocols followed local animal ethics regulations and were approved by the Institutional Animal Care and Use Committee of National Yang-Ming University (Approval No. 1021218).

### Histological analysis

Skeletal muscle samples, soleus, gastrocnemius and femoris, were collected separately and fixed with 10% formalin buffered with phosphate. They then underwent tissue processing and were embedded in paraffin. Finally, Haemotoxylin and Eosin (H&E) staining of tissue sections (3–4 μm) were carried out using standard protocols.

### Transmission electron microscopy (TEM)

The muscle tissue samples from the soleus and gastrocnemius were fixed separately in a mixture of 1.5% glutaraldehyde and 1.5% paraformaldehyde in 0.1 M cacodylate buffer at pH 7.3, and then post-fixed in 1% OsO_4_ and 1.5% potassium hexacyanoferrate. Next the samples were rinsed in cacodylate, and 0.2 M sodium maleate buffers (pH 6.0) followed by block-stained with 1% uranyl acetate. After dehydration, the skeletal muscle samples were embedded in Epon (14120, EMS) and sectioned for TEM as described previously [25].

### RNA sequencing and analysis

Total RNA was isolated from muscle tissue samples using TRIzol Reagent (Thermo Fisher Scientific, Waltham, MA, USA). The quality of total RNA was tested using an Agilent 2100 Bioanalyzer (Agilent Technologies, Palo Alto, CA, USA); samples with a RNA Integrity Number (RIN) of greater than 8 were subjected to RNA sequencing. The RNA sequencing was performed on a NextSeq 550 platform by National Yang-Ming University VYM Genome Research Center. Counts were estimated by the rsem R package using raw fastq files; this filtered out genes that did not have an appropriate count, which was defined as having more than 150 counts detected in at least half of the samples. The PCA plot was generated by MetaboAnalyst (https://www.metaboanalyst.ca) using the filtered counts. Next, the differentially expressed genes (DEGs) were pinpointed using the DESeq2 R package; these had an FDR <0.1 (*p* < 0.005). The workstream is presented in Supplementary Figure 1. The differentially expressed genes (DEGs) calculated from the RPKM (reads per kilobase of exon model per million reads) in RNA-Seq data were annotated using the IPA approach (Ingenuity Systems^®^, http://www.ingenuity.com/) and Go enrichment analysis (http://pantherdb.org/) [26].

**Figure 1:**
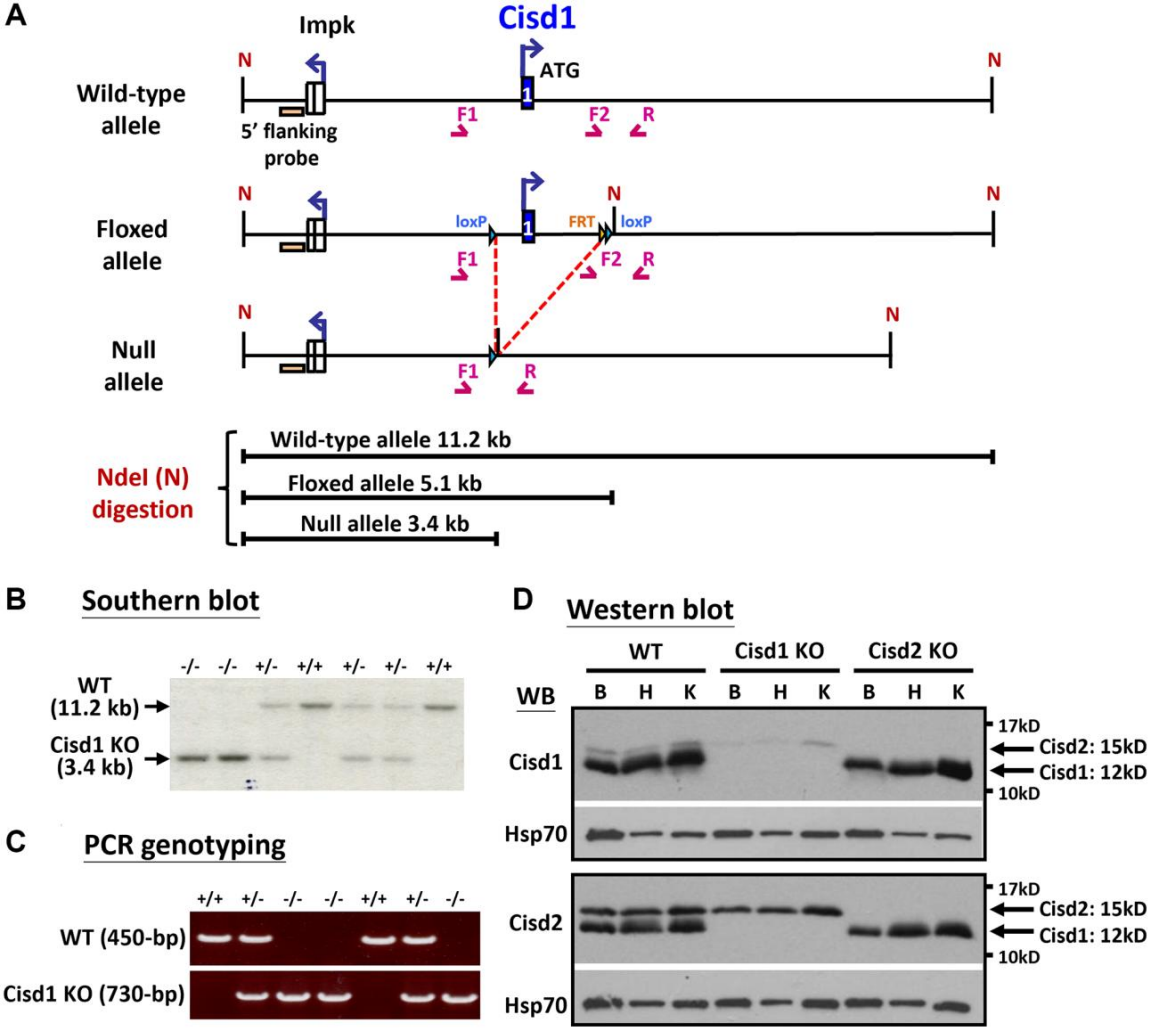
**Generation of the Cisd1 knockout (KO) mouse model.** (**A**) The process of generating the CIsd1 knockout mouse. (**B**) Southern blot analysis. Genomic DNA was digested by Nde I and probed with a 5’ flanking probe. (**C**) The genotyping results are able to identify wild-type, heterozygous and homozygous knockout mice. The primers for PCR genotyping are F1: 5’-GTGTGAGTGTGTCTGTACCTAG-3’, F2: 5’-CTGTCTACATCAGAGCAGAACAC-3’ and R: 5’-CAACTGCACCACAAATCATGTGAG-3’. (**D**) Protein expression of Cisd1 and CIsd2 in wild-type (WT), Cisd1 KO and Cisd2 KO mice. Abbreviations: B: brain; H: heart; K: kidney. Mouse age, 4–5 weeks old.

### Statistical analysis

The data of survival rate was presented as mean value and calculated using Log-rank Mantal-Cox tests (Prism 8.0). The data of other experiments was presented as mean ± SD. Comparison between two groups was carried out using the two-tailed Student *t*-test. *P* < 0.05 was considered significant when analyzing statistical differences among groups. For body weight, we evaluated the data using the Shapiro-Wilk normality test, which is more suitable for small sample sizes (*N* < 50). The results indicated that samples in some groups did not follow a normal distribution (*p* < 0.05 using the Shapiro-Wilk normality test). Therefore, we re-analyzed the body weight data using the Kruskal-Wallis test with Dunn’s multiple comparisons test, and the statistical results are presented in Supplementary Table 1. For muscle fiber size, the sample size was too small for the Shapiro-Wilk normality test. Consequently, we re-analyzed the data using the Kruskal-Wallis test with Dunn’s multiple comparisons test to identify any statistical differences.

## RESULTS

### Cisd1 and Cisd2 double knockout (DKO) mice had more severe phenotypes

**Figure 2:**
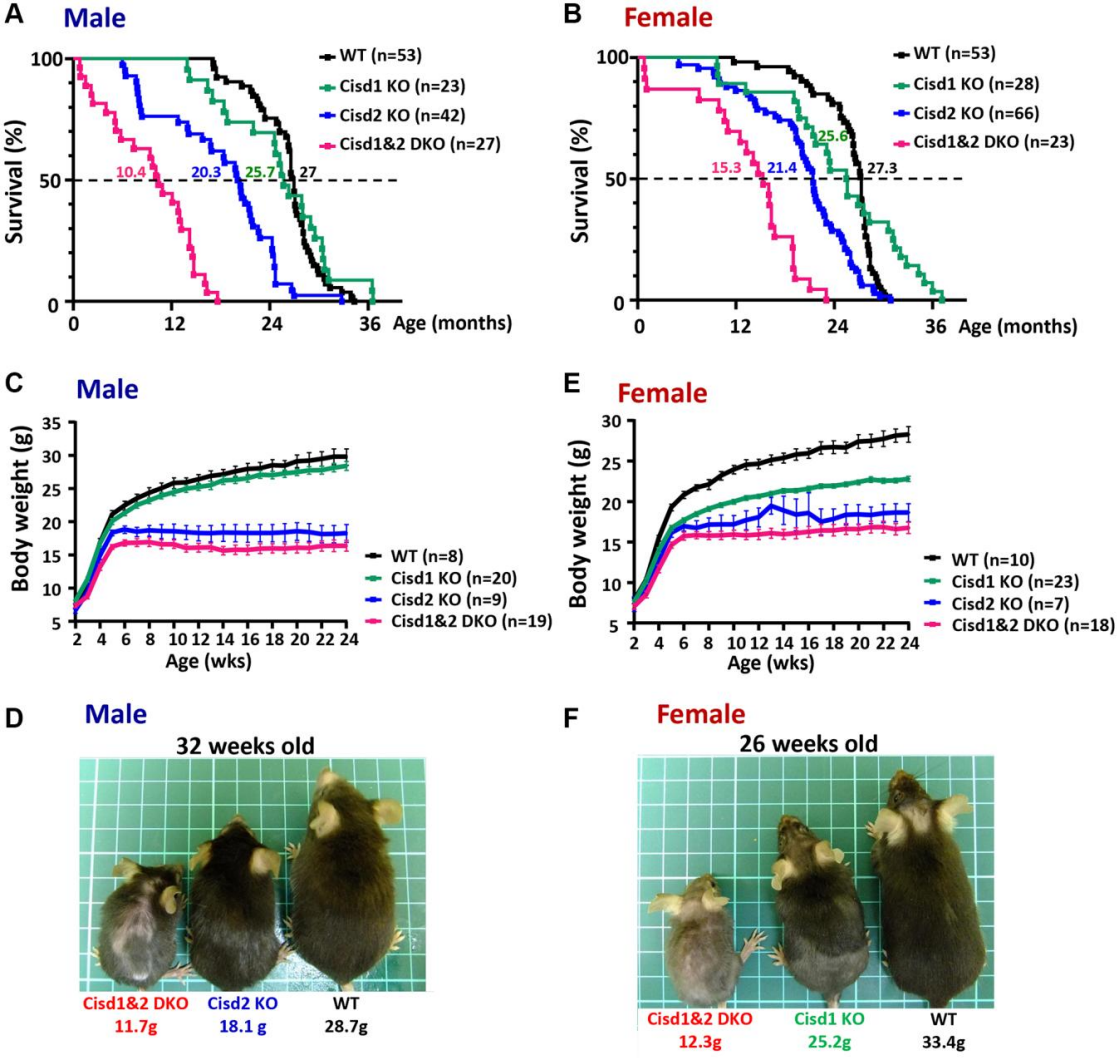
**Shortened lifespan and severe growth retardation in the Cisd1&2 DKO mice.** (**A**, **B**) The lifespan of the Cisd1 KO, Cisd2 KO and Cisd1&2 DKO male (**A**) and female (**B**) mice on B6/129 mixed background. Median survival of each genotype is present in the graph. Statistical analyses were calculated using Log-rank Mantal-Cox tests (Prism 8.0) and *p* < 0.001 (except WT vs. Cisd1 KO). (**C**) The growth curves of the male Cisd1 KO, Cisd2 KO and Cisd1&2 DKO mice. Body weight was monitored weekly from 2 weeks to 24 weeks old. The body weight of the Cisd2 KO and Cisd1&2 DKO mice were lower than WT mice from 4–5 weeks old, whereas the body weight of Cisd1 KO mice showed no obvious decrease compared to the WT mice. Data are shown as mean± SEM. (**D**) The photographs were of Cisd2 KO, Cisd1&2 DKO and WT male mice at 32 weeks of age. This photograph was taken from the dorsal view. (**E**) The growth curves of the female Cisd1 KO, Cisd2 KO and Cisd1&2 DKO mice. Body weight was monitored weekly from 2 weeks to 24 weeks old. The body weight of Cisd1 KO, Cisd2 KO and Cisd1&2 DKO mice were less than WT mice from 4 weeks old. Data was shown as mean± SEM. (**F**) The photographs were of Cisd2 KO, Cisd1&2 DKO and WT female mice at 26 weeks of age. These photographs were taken from the dorsal view.

**Figure 3:**
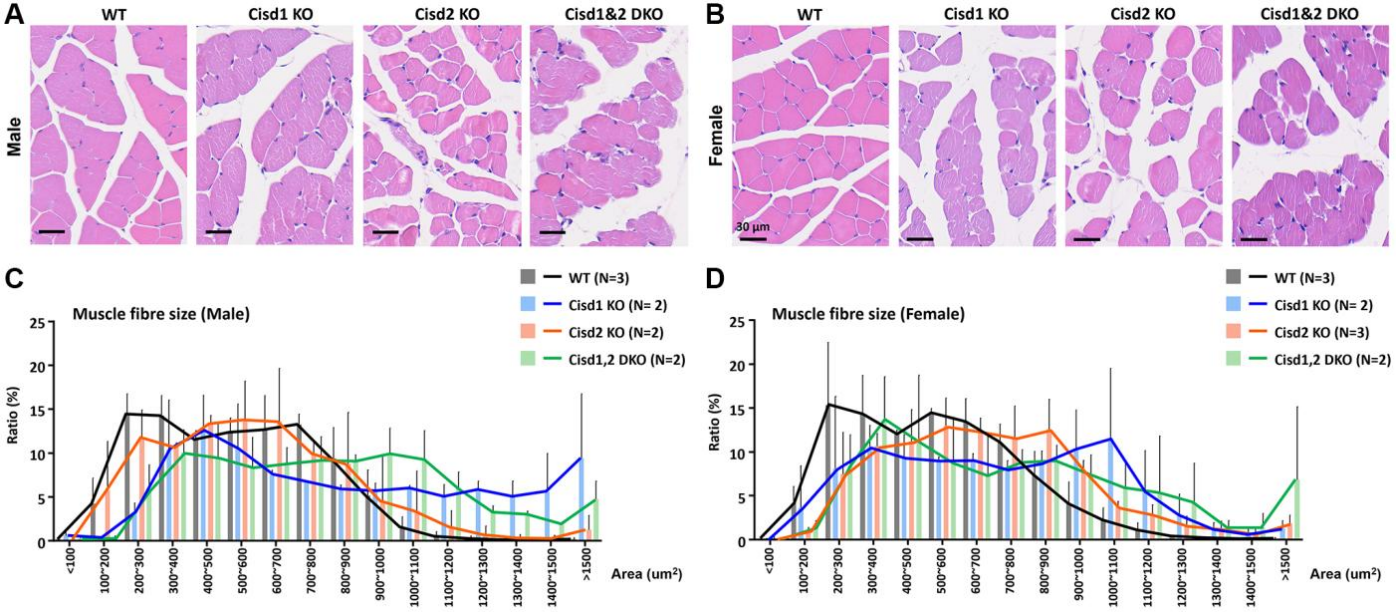
**Histological analysis of skeletal muscle in the Cisd1&2 DKO mice.** (**A**, **B**) H&E staining analysis of skeletal muscle (femoris) in Cisd1 KO, Cisd2 KO and Cisd1&2 DKO mice at 5 weeks old. Scale bar, 30 μm. (**C**, **D**) Histograms of muscle fiber size in male and female Cisd1 KO, Cisd2 KO and Cisd1&2 DKO mice. Data was shown as mean± SD. In (**C**, **D**), Kruskal-Wallis test with Dunn’s multiple comparisons test was performed to analyze the statistical differences among genotypes and did not find the statistical difference.

Our previous report indicated that Cisd2 KO mice showed growth retardation, weakened skeletal muscles and a short lifespan [12, 18]. Cisd1 and Cisd2 belong to the same protein family and also have the identical domains, the transmembrane domain and the CDGSH iron sulfur domain [27]; therefore, whether the ablation of both Cisd1 and Cisd2 has a synergistic effect on skeletal muscle defects is an open question. We generated the Cisd1 KO mice as shown in Figure 1A. Genotype analysis was carried out by Southern blot and PCR (Figure 1B, 1C), and the Cisd1 protein was found to be completely deleted in the Cisd1 KO mice (Figure 1D). The survival rate of Cisd2 KO mice was lower than the WT and Cisd1 KO mice for both genders; however, the survival rate of DKO mice was lower than the three other groups (Figure 2A, 2B). Cisd1&2 DKO and Cisd2 KO mice showed growth retardation for both genders and showed early aging as shown in the photographs shown (Figure 2C–2F). This was obviously from 5 weeks old. Notably, Cisd1 deletion led to growth retardation in female but not in male mice, which is also shown in the photographs (Figure 2E, 2F). The probability value (*p*-value) across the four groups of mice at different ages are presented in Supplementary Table 1. Mild pathological defects were identified in the muscle sections from Cisd1 KO mice. Some muscle fibers exhibited slight hypertrophy, and a few showed losses of homogeneity of the sarcoplasm (Figure 3A, 3B). Partial muscle degeneration and pathological defects, such as fiber shrinking, vacuolated cytoplasm, and distortion of muscle fibers, were observed in Cisd2 KO mice (Figure 3A, 3B). More severe muscle defects were noted in muscle sections of DKO mice at 5 weeks old, including loss of homogeneity in the muscle cytoplasm, distortion of fiber boundaries, and altered and/or variable fiber sizes (Figure 3A, 3B). An increased number of larger muscle fibers were found in Cisd1 KO and DKO mice, particularly in male mice (Figure 3C). Additionally, in males, larger muscle fibers (>1000 μm²) averaged 36.7% in the Cisd1 KO and 28.4% in the DKO mice, compared to 7.5% in the Cisd2 KO and 2.6% in the WT mice. In females, larger muscle fibers (>1000 μm²) averaged 23.0% in the Cisd1 KO and 24.8% in the DKO mice, compared to 12% in the Cisd2 KO and 3.8% in the WT mice (Figure 3D). We also quantified the number of muscle fibers with central nuclei. The ratios of muscles with central nuclei in Cisd1 KO, Cisd2 KO, and DKO mice were comparable to those in WT mice, as shown in Supplementary Figure 2. Thus, it was clear that the genetic defects of Cisd1 and Cisd2 had synergistic effects and led to more severe phenotypes in skeletal muscle; however, muscle degeneration in our KO mice is unlikely to be caused by intense cycles of degeneration and regeneration. Remarkably, the body weight of the Cisd1 KO female mice was significantly lower than WT female mice, but this phenotype was not observed in the Cisd1 male, indicating that the mild muscle defects in the Cisd1 KO male mice might not seem to contribute to weight loss.

### Mitochondrial defects, myofibril degeneration, ER stress and Golgi degeneration showed increased presence in Cisd1&2 DKO mice

**Figure 4:**
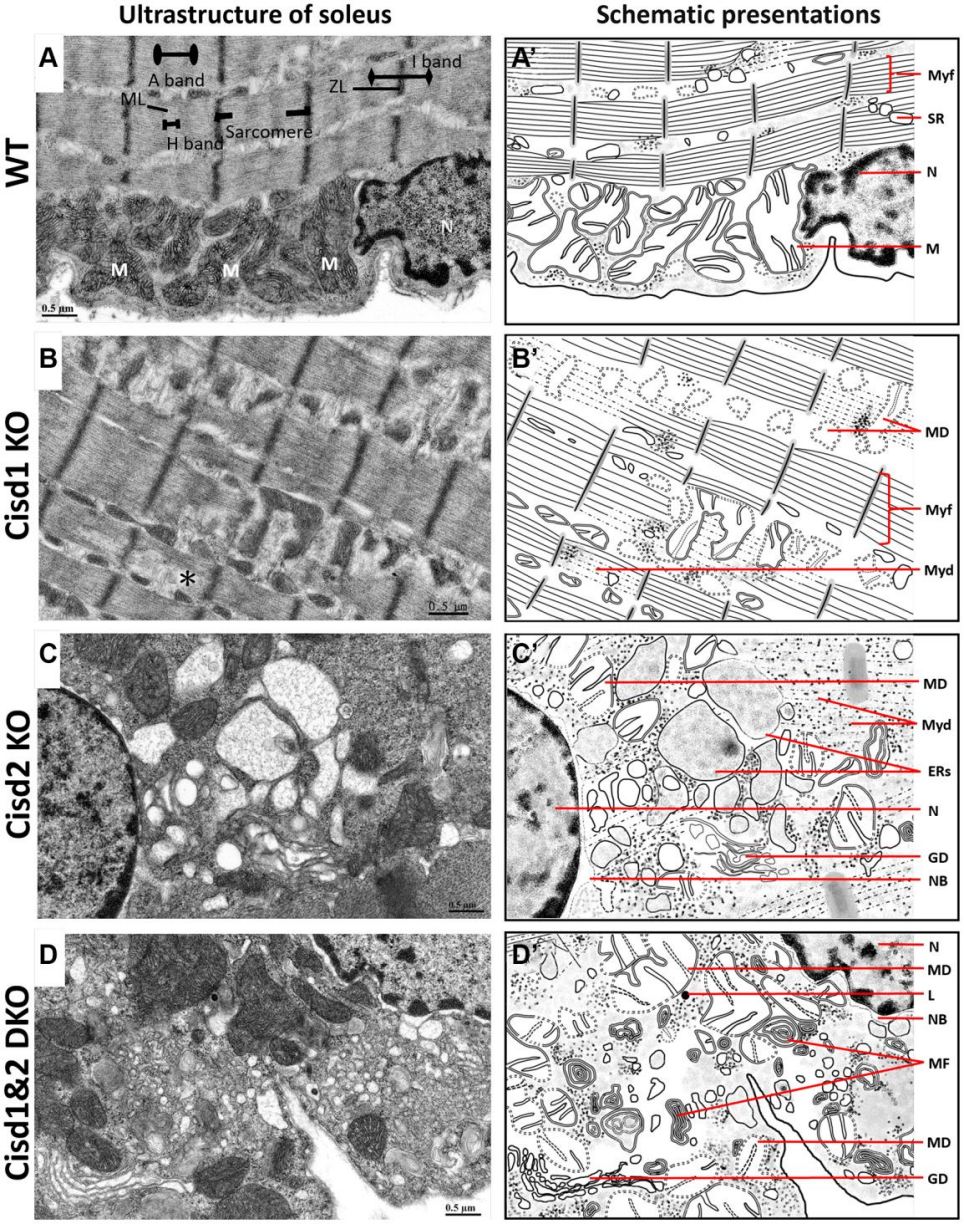
**Ultrastructure of skeletal muscle (soleus) from Cisd1&2 DKO mice.** (**A**) Architecture of the soleus in WT mice. ZL, Z line; ML, M line; M, mitochondria; N, nucleus. (**B**) Mitochondrial defects, myofibril degeneration (*) and ER stress in Cisd1 KO soleus. (**C**) Mitochondrial defect, myofibril degeneration, necrosis and ER stress in Cisd2 KO soleus. (**D**) Mitochondrial defect, myofibril degeneration, necrosis and ER stress in Cisd1&2 DKO soleus. (**A**–**D**) were shown at higher magnification (yellow squares) in Supplementary Figure 3. (**A’**–**D’**) are schematic presentations of the ultrastructure of the soleus shown in (**A**–**D**). Abbreviations: Ne: Nucleolus; NB: Nuclear envelope breakdown; MD: Mitochondrial defect; Myf: Myofibril; Myd: Myofibril degeneration; Ers: ER stress; ER: Endoplasmic Reticulum; SR: Sarcoplasmic Reticulum; Avi: Autophagosome; Avd: Autolysosome; L: Lysosome; MF: Myelin Figure; GD: Golgi degeneration. Mouse age, 5 weeks old.

**Figure 5:**
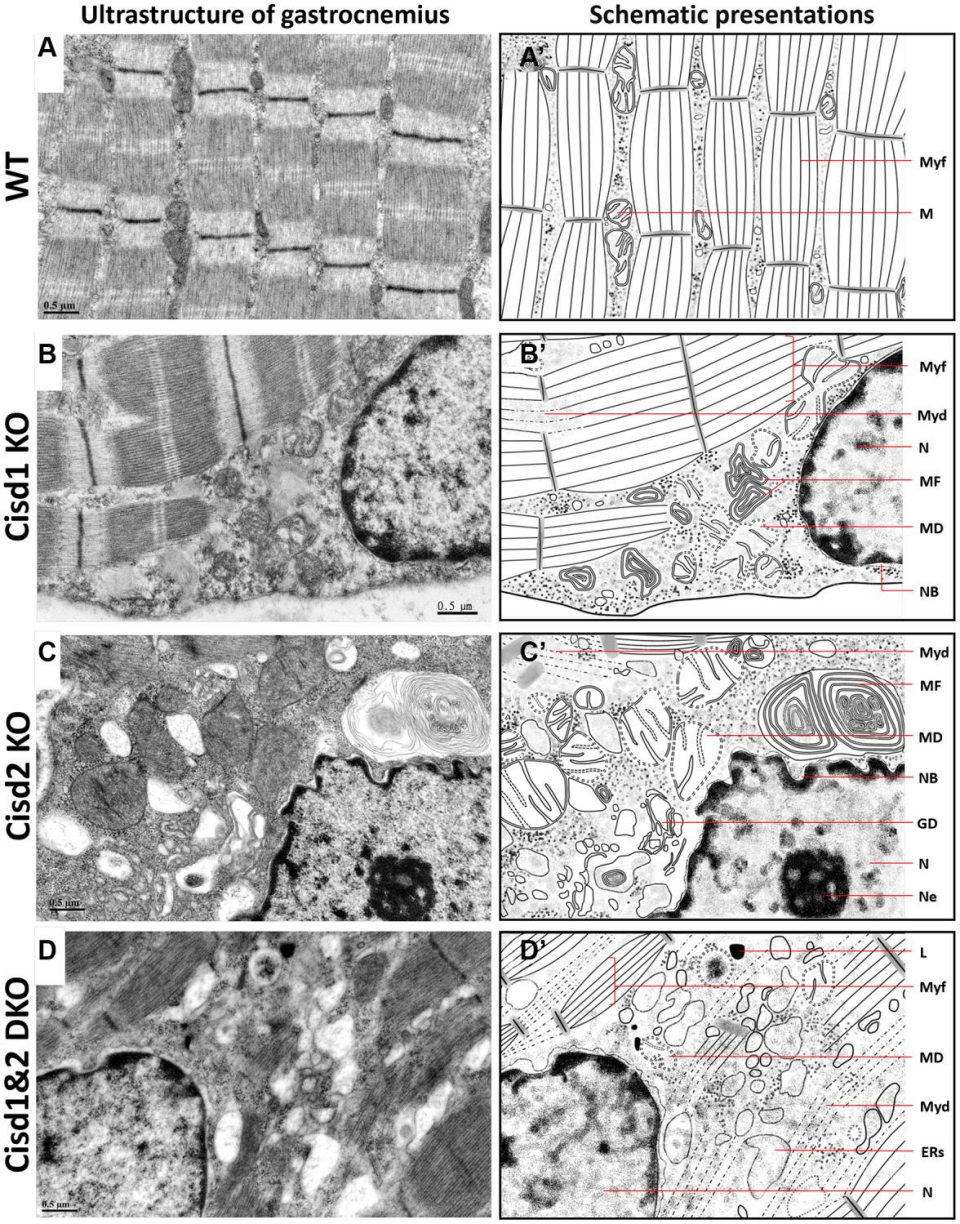
**Ultrastructure of skeletal muscle (Gastrocnemius, Gas.) in Cisd1&2 DKO mice.** (**A**) Architecture of soleus in WT mice. (**B**) Mitochondrial defects, necrosis and ER stress in Cisd1 KO soleus. (**C**) Mitochondrial defects, necrosis and ER stress in Cisd2 KO soleus. (**D**) Mitochondrial defects, myofibril degeneration, necrosis and ER stress in Cisd1&2 DKO soleus. (**A**–**D**) were shown at higher magnification (yellow squares) in Supplementary Figure 4. (**A’**–**D’**) are schematic presentations of the ultrastructure of the gastrocnemius shown in (**A**–**D**). Abbreviations: Ne: Nucleolus; NB: Nuclear envelope breakdown; MD: Mitochondrial defect; Myf: Myofibril; Myd: Myofibril degeneration; ERs: ER stress; ER: Endoplasmic Reticulum; L: Lysosome; MF: Myelin Figure; GD: Golgi degeneration. Mouse age, 5 weeks old.

To examine the cellular defects in skeletal muscles of these genetically defect mice, TEM was performed in order to observe the ultrastructure of soleus and gastrocnemius at 5 weeks old. The Cisd1 KO soleus showed mild mitochondrial defects (MD) and myofibril degeneration (MyoD); however, the Cisd2 KO soleus and Cisd1&2 DKO soleus had additional phenotypes, necrosis, and ER stress, besides MD and MyoD (Figure 4A–4D, 4A’–4D’ and Supplementary Figure 3). The Cisd1 KO and Cisd2 KO gastrocnemius showed MD, necrosis, and ER stress, while the Cisd1&2 DKO gastrocnemius showed more severe defects, including MD, ER stress, necrosis and MyoD (5A–5D, 5A’–5D’ and Supplementary Figure 4). In addition, nuclear envelope breakdown and Golgi degeneration were also found in the Cisd2 KO muscles, and these were more severe in Cisd1&2 DKO muscles (Figures 4C, 4D and 5C, 5D). Dysfunction of cellular organelles, such as the ER and Golgi apparatus, led to the errors in protein translation, modification, and transports, eventually resulting in cell death. As expected, the deletion of both Cisd1 and Cisd2 had the synergistic effects on various process and was able to induce premature aged phenotype as well as cell death.

### The changes in RNA expression patterns in Cisd1&2 DKO mice

**Figure 6:**
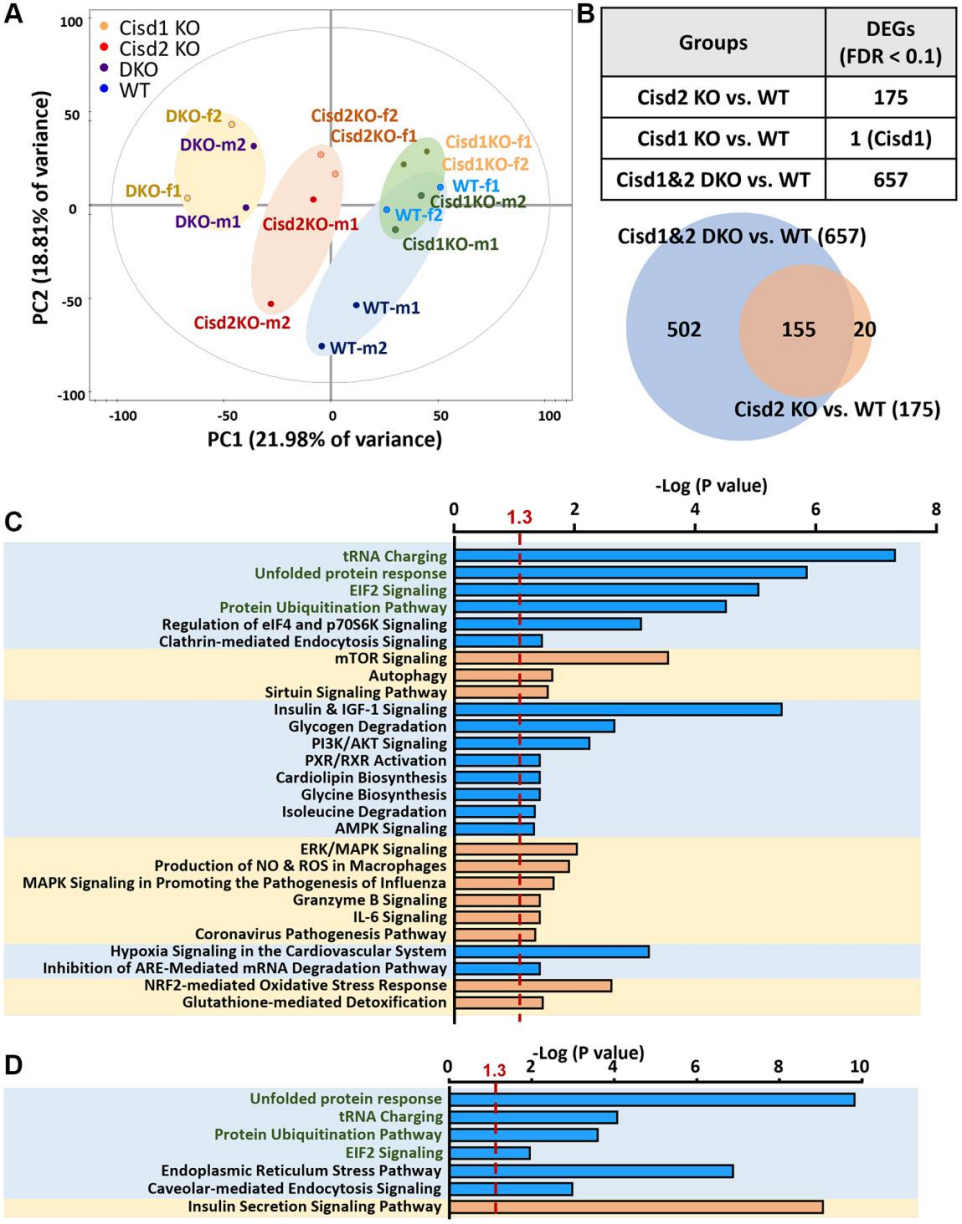
**Gene expression profiling of skeletal muscle using RNA sequencing analysis.** (**A**) The RNA expression pattern analysis in skeletal muscle (femoris) using Principal Component Analysis (PCA). A total of 5977 genes were involved in this analysis. Two samples in each group and genders were used. Mouse age, 5 weeks old. (**B**) Analysis of differently expressed genes (DEGs) between different groups. 155 genes were observed in both groups, DKO vs. WT and Cisd2 KO vs. WT. (**C**) IPA analysis of gene expression in DKO muscles alone (502 genes). (**D**) The IPA analysis of gene expression in both of DKO and Cisd2 KO muscles (155 genes). The X axis represents negative log *p*-values based on the probability that genes in the uploaded dataset were included in the predefined IPA canonical pathways. Pathways with green words were the pathways which were found in both analyses in (**C**, **D**).

**Figure 7:**
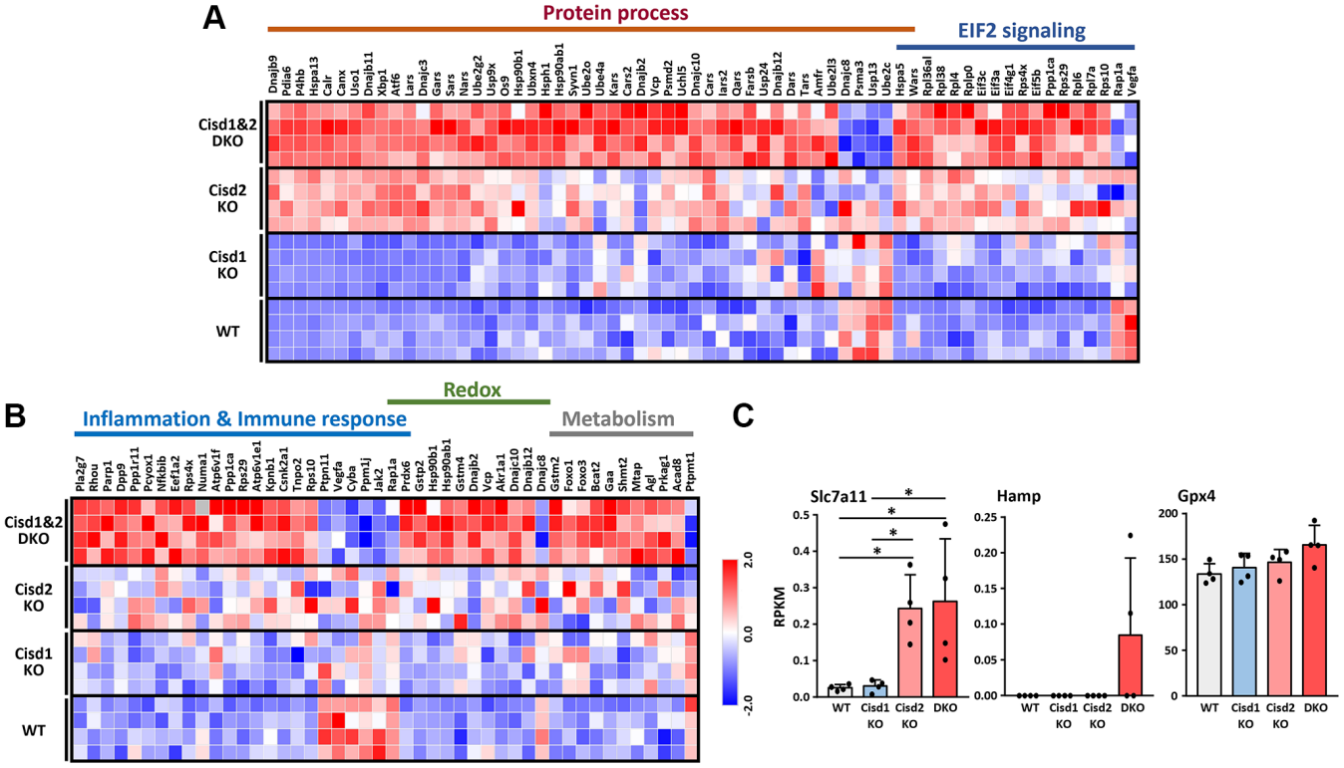
**RNA sequencing data in skeletal muscles presented as a heatmap.** (**A**) Heatmap analysis of genes in the pathways showed significant changes in both CIsd2 KO and Cisd1&2 DKO muscles. (**B**) Heatmap analysis of genes in the pathways showed significant changes in Cisd1&2 DKO muscles alone. (**C**) The expression levels of genes that are involved in programmed cell death, ferroptosis. Data are presented as mean ± SD. ^*^p < 0.05. The data were analyzed by one-way ANOVA and the Turkey *post hoc* test.

To understand the molecular mechanisms that are involved in the synergistic pathological process present in Cisd1&2 DKO mice, RNA sequencing analysis were performed on skeletal muscles with Cisd1 and/or Cisd2 gene deficiency. The PCA analysis showed four distinct clusters, WT, Cisd1 KO, Cisd2 KO and Cisd1&2 DKO. Cisd1 KO samples were clustered together and close to the WT samples. Cisd2 KO samples were also clustered together and further away from the WT samples. Finally, the Cisd1&2 DKO samples were also clustered and at the greatest distance from the WT samples (Figure 6A). The number of DEGs between Cisd2 KO and WT groups were 175 genes and between Cisd1&2 DKO and WT groups there were 657 genes; additionally, 155 genes were found in both sets of data (Figure 6B). The heatmaps of gene expression in both Cisd2 KO and Cisd1&2 DKO muscle (155 genes), in Cisd2 KO muscle alone (20 genes) and in the Cisd1&2 DKO muscle (502 genes) revealed there were significant changes of gene in expression across the different mouse groups (Supplementary Figure 5A–5C). Totals of 155 genes and 502 genes were separately mapped using IPA software (Figure 6C, 6D). In the top scoring canonical pathways produced by the IPA analysis, four pathways were identified, namely the unfolded protein response, tRNA charging, protein ubiquitination and EIF2 signaling; the genes involved in these pathways are presented in a heatmap (Figure 7A). The functional enrichment analysis results identified a number of significant Gene Ontology (GO) terms in Cisd1&2 DKO and Cisd2 KO muscles (Supplementary Figure 5D, 5E). Most of these GO terms were highly related to protein processing, including the initiation of translation, translational processes, modification, transport, and protein degradation. Except for protein process and EIF2 signaling, several critical pathways were significantly changed in Cisd1&2 DKO muscle sample and these functions are related to inflammation and immune response, redox reactions, and metabolism (Figure 7B). Finally, alterations in signaling and biological process that induce programmed cell death, such as ferroptosis (Figure 7C), as well as the non-regulated cell death, and necrosis were pinpointed (Figures 4 and 5). Solute Carrier Family 7 Member 11 (SLC7A11) is a critical regulator of ferroptosis and a key component of the cystine-glutamate antiporter. SLC7A11 was upregulated in Cisd2 KO and Cisd1&2 DKO mice. Additionally, hepcidin antimicrobial peptide (Hamp) and glutathione peroxidase 4 (Gpx4), which are closely related to ferroptosis, would seem to be upregulated in Cisd1&2 DKO mice.

**Figure 8:**
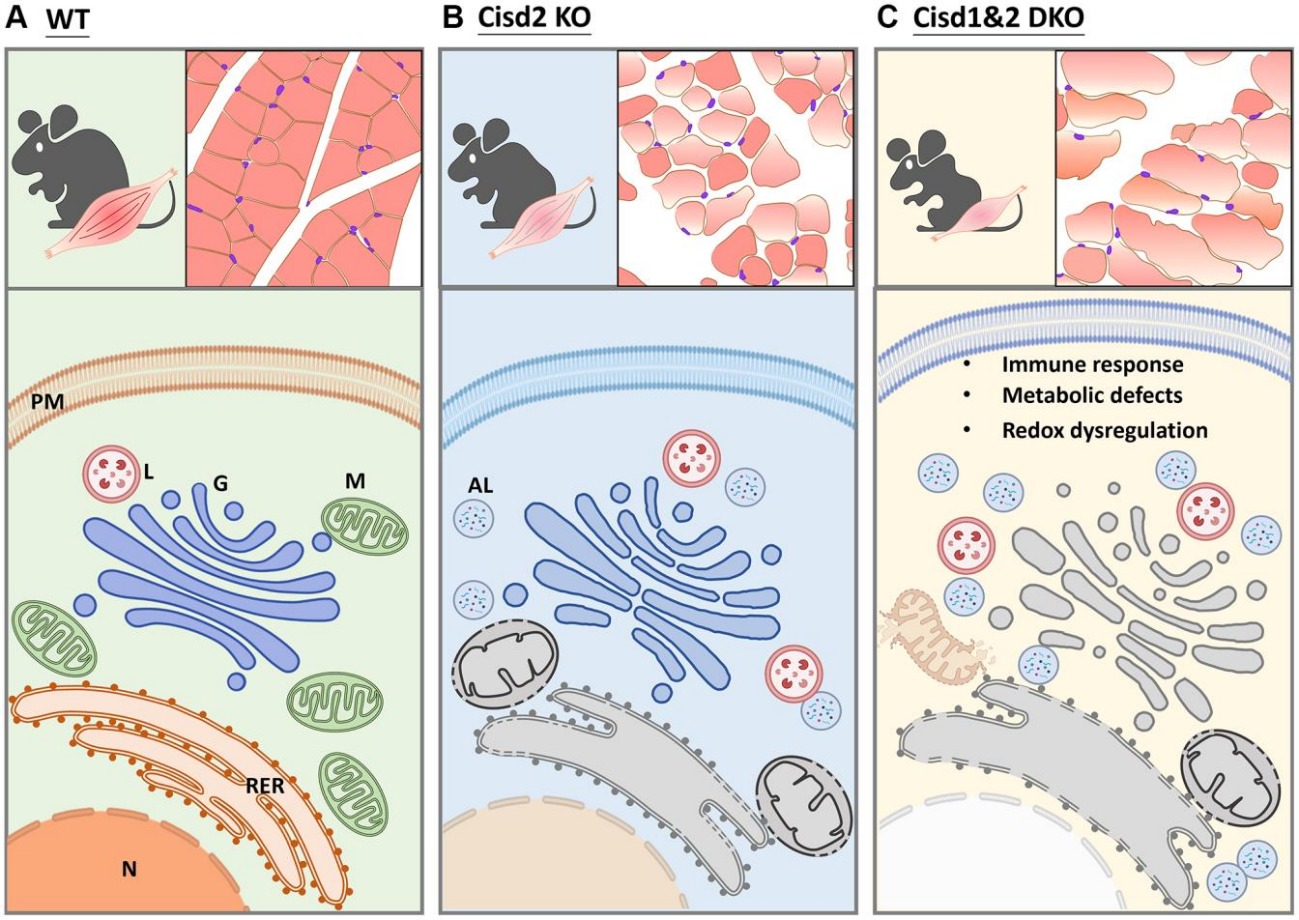
**Schematic showing the alteration in biological processes identified in Cisd2 KO and Cisd1&2 DKO mice.** (**A**) In wild type (WT) mice, the protein process, including translation, transportation, modification and degradation, is well-controlled by functional organelles. (**B**) Cisd2 deletion leads to organelle degeneration and autolysosome formation. (**C**) Ablation of both Cisd1 and Cisd2 has a synergistic effect that results in organelle breakdown and cell death.

To sum up, the ablation of Cisd1 and Cisd2 causes mitochondrial dysfunction, ROS generation and ER stress, which creates defects in protein translation and modifies the ER, as well as affecting protein transport from ER to Golgi apparatus (Figure 8). As mentioned earlier, the Cisd1 deletion mouse results in a milder phenotype in skeletal muscle; however, when a mouse lacks both Cisd1 and Cisd2 expression, the synergistic effects exacerbate the phenotypic consequence in muscle.

## DISCUSSION

During the analysis of the mouse phenotypes, the Cisd1&2 DKO mice were found to have the lowest survival rate as well as the lowest body weight compared to the Cisd1 and Cisd2 mice. This lower body weight was due to a loss of skeletal muscle and fat based on the gross phenotype and the histological findings. Ablation of Cisd2 in muscle led to muscle defects such as fiber shrinking, vacuolated cytoplasm, distortion of muscle fibers, myofibril degeneration, organelle damage and cell death. On the other hand, Cisd1 deletion seems to expatiate the muscle defects present in Cisd2 KO mice. Our sequencing results clearly showed that Cisd1 KO muscles had no significant difference compared to WT muscles, while Cisd2 deletion led to alterations in several signaling pathways and biological processes. Interestedly, the changes in additional pathways and mechanisms, including immune responses, redox regulation, and metabolism, occurred in Cisd1&2 DKO muscles, neither in Cisd2 KO muscles nor in Cisd1 KO muscles. The aforementioned information seems to support the hypothesis that Cisd1 and Cisd2 have synergistic effects on signaling pathways as well as various molecular mechanisms found in skeletal muscles. Cisd2 dysfunction alone disrupts MAM stability, while Cisd1&2 DKO exacerbate mitochondrial damage and ER stress, which the leads to programmed cell death.

### Defects affecting organelles

During the process of protein synthesis, including translation, modification, folding, transportation, and degradation, our previous reports have provided evidence that Cisd2 deletion contributes to ER stress and disrupts calcium homeostasis. Indeed, in this study, Cisd2 KO and Cisd1&2 DKO resulted in upregulated X-box binding protein 1 (Xbp1) and activating transcription factor 6 (ATF6) expression, both of which are the critical molecules in ER stress [28, 29]. In Cisd2 KO and Cisd1&2 DKO muscles, a group of genes involved in molecular chaperoning were upregulated, including DnaJ heat shock protein family (Hsp40) (Dnaj), Heat shock protein (Hsp), beta-subunit of prolyl 4-hydroxylase (p4hb), Calreticulin (Calr), and Calnexin (Canx), which are responsible for suppressing aggregation and shuttling of misfolded proteins for degradation [30–32]. Calr and Canx act as adaptors to recruit other ER chaperones to assist in different aspects [33]. If protein folding fails, the terminal misfolded proteins are transported for degradation via the ER associated protein degradation (ERAD) pathway. Several related genes to the above are also upregulated in Cisd2 KO and Cisd1&2 DKO muscles, that in the ubiquitin-associated mechanisms of protein degradation [34, 35]. In this situation, cells that are unable to produce functional proteins and initiate compensatory effects to start translation. As a result of this, most amino acid-tRNA synthetases were upregulated, and eIF2 signaling was also activated. Moreover, Cisd1&Cisd2 DKO mice had severe mitochondrial defects in their skeletal muscles. Several genes participating in muscle energy homeostasis and mitochondrial metabolism were found to be upregulated in the double knockout mice, such as Forkhead box class O 1 (Foxo1), Forkhead box class O 3 (Foxo3), Serine hydroxymethyltransferase 2 (Shmt2), Protein kinase AMP-activated non-catalytic subunit gamma 1 (Prkag1), and Protein tyrosine phosphatase mitochondrial 1 (Ptpmt1) [36–40]. Recent studies have indicated that Cisd expression levels are highly associated with mitophagy regulation [41, 42]. In Drosophila and in a human cell line, loss of Cisd (majorly Cisd1) interrupts the process of mitophagy and damaged mitochondria then accumulate in cells, finally triggering cell death. This may be one possibility for explaining why loss of Cisd1 exacerbates the phenotypes of Cisd2 KO mice. Both ER stress and mitochondrial damage directly or indirectly reduce MAMs stability, and all of the above will contribute the process of cell death.

### Inflammation and oxidative stress

The alterations in several critical signaling pathways and mechanisms were only observed in the Cisd1&Cisd2 DKO muscles. Most of them are related to microenvironmental changes, such as inflammation, redox regulation (antioxidant capacity) and metabolic changes. Dipeptidyl peptidase 9 (Dpp9) was highly upregulated in Cisd1&Cisd2 DKO muscles. Dpp9/8 inhibition would activate the inflammasome sensor Nlrp1b resulting in procaspase-1-induced cell death [43]. Dpp9 acts as a multifunctional inflammasome regulator and is able to induce a lytic form of cell death, pyroptosis, in monocytes and macrophages [44]. Interestedly, MAMs are important to inflammasome activation, including the NOD-like receptor protein 3 (NLRP3)-mediated inflammasome [45]. Several genes involved in redox signaling show changes in Cisd1&Cisd2 DKO muscles, including Aldo-keto reductase family 1 member A1 (Akr1a1), Peroxiredoxin 6 (Prdx6), and different distinct classes of glutathione S-transferases. As previous reports indicated, Akr1a1 participates in redox-based cellular signaling and Prdx6 is involved in the nuclear factor erythroid 2-related factor 2 (Nrf2)-mediated antioxidant defense system [46, 47]. The dysregulation of inflammasomes and the presence of increased oxidative stress then destroy cellular homeostasis thus accelerating programmed cell death.

### Limitation and prospectives

In this study, we demonstrated that Cisd2 is more essential than Cisd1 for maintaining skeletal muscle function and morphology in mice. Cisds gene deletion had huge influence on protein process and degradation; this results eventually in cell damage followed by cell death. Signaling regulation in our Cisds gene deleted models pinpoints critical mechanisms involved in modulating crosstalk and homeostasis among various organelles in cells. Additionally, immune response and metabolic regulation exacerbate muscle defects as the mice age. Intracellular defects typically induce the release of factors that circulate throughout the body, and this will lead to compensatory effects or disruption of the functioning of other cells or tissues. As a consequence of this, mice exhibit premature aging features, a retardation of growth, and a shortened lifespan. Every tissue and organ are able to release signals that modulate other tissues and organs, meaning critical aging-related factors may be secreted from both skeletal muscle and non-muscle tissues. Understanding the regulation of crosstalk between tissues will help to facilitate insights into the mechanisms involved in systemic aging processes.

Cisds seems to be a critical target when investigating muscle disorders and this may lead to novel therapeutic strategies. Our studies have shown that Cisd2 deletion leads to more severe physiological phenotypes compared to Cisd1 deletion, suggesting that Cisd2 may play a more important role than Cisd1. Previous research has demonstrated that pharmacological enhancement of Cisd2 expression can mitigate aging-related defects in the skin [48]. Therefore, there is a significant potential to rescue muscle defects by elevating Cisd2 expression.

## Supplementary Materials

Supplementary Figures

Supplementary Table 1
