# Supplementary material for: Cisd1 synergizes with Cisd2 to modulate protein processing by maintaining mitochondrial and ER homeostasis
Source: Aging (Albany NY). 2025 May 8;17(5):1275–97. doi: 10.18632/aging.206249 (PMC12151500; doi:10.18632/aging.206249)
Supplement: Supplementary Figures [file aging-17-206249-s001.pdf]

SUPPLEMENTARY FIGURES

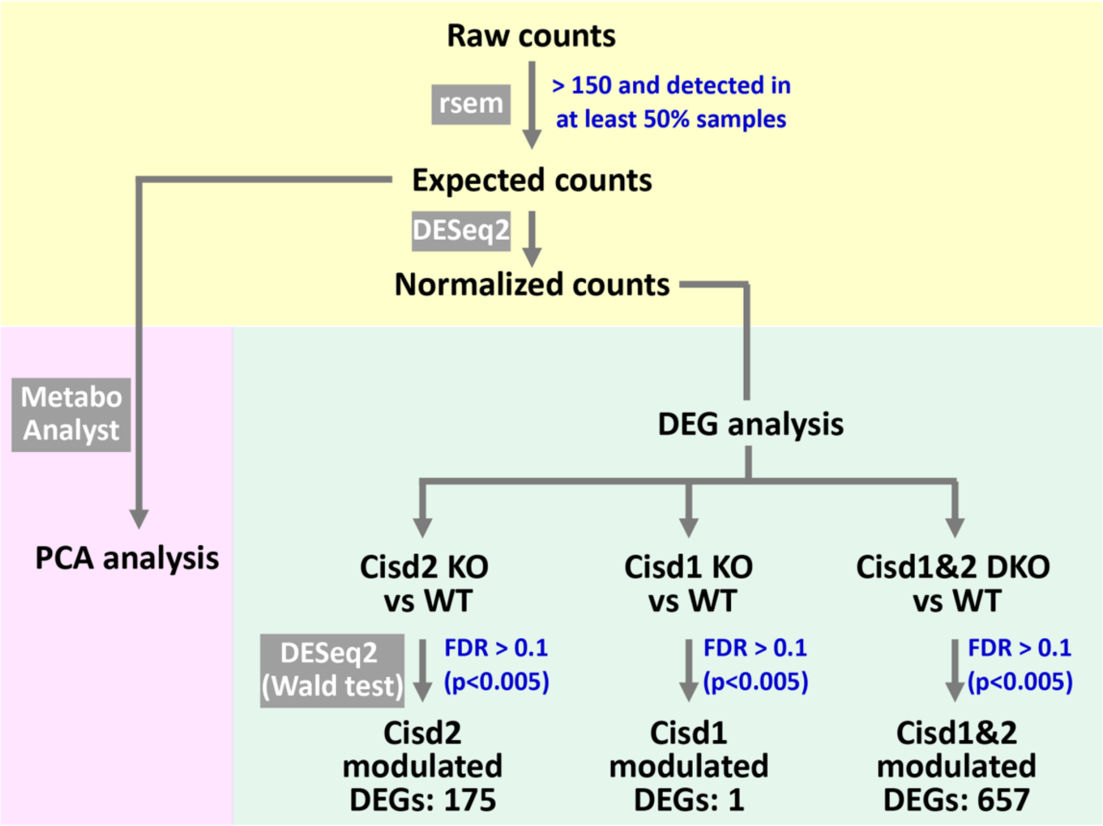

Supplementary Figure 1. Workflow of RNA-sequencing analysis. Expected counts defined as morethan 150 counts in at least 50% of samples were detected using rsem R package. For PCA analysis, expected counts were subjected to MetaboAnalyst. For differential expression gene (DEG) analysis, normalized counts in Cisd1 KO, Cisd2 KO, and DKO samples were separately compared with WT sample using Wald test in DEseq2 R package. DEG was defined as FRD <0.1 ($p < 0.005$).

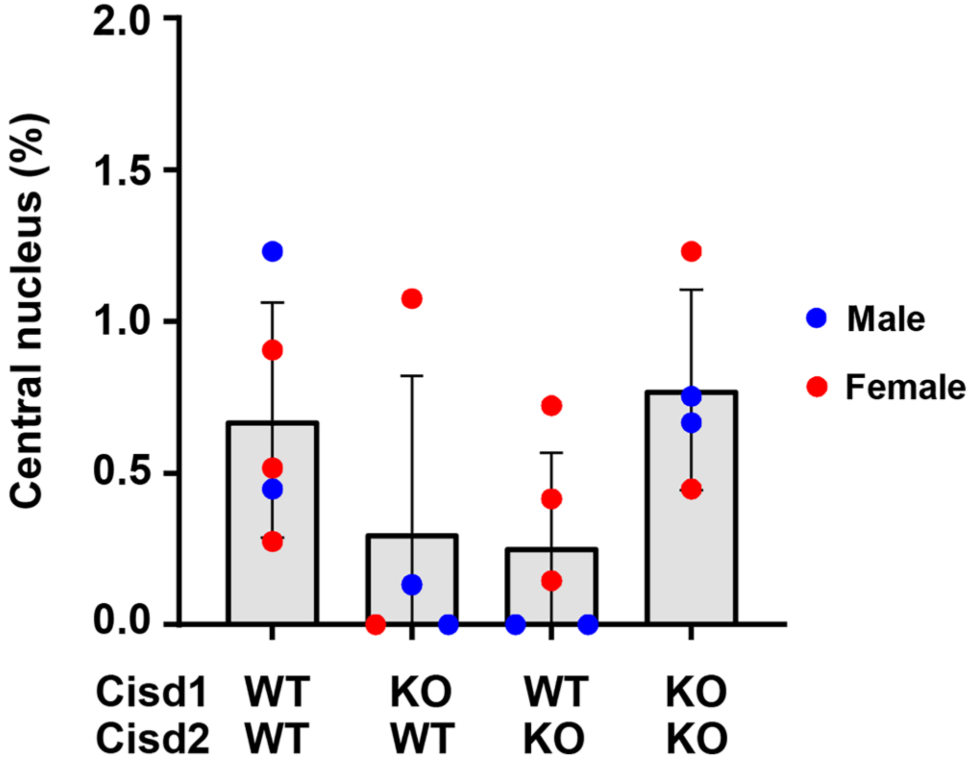

Supplementary Figure 2. Quantitation of muscle fiber with a central nucleus in skeletal muscle of the Cisd1&2 DKO mice. Central nucleus was manually identified and counted in H&E staining images of skeletal muscle (femoris) in Cisd1 KO, Cisd2 KO and Cisd1&2 DKO mice at 5 weeks old. Data was shown as mean ± SD. Kruskal-Wallis test with Dunn's multiple comparisons test was performed to analyze the statistical differences among genotypes and no statistical difference was found in any pair of comparison.

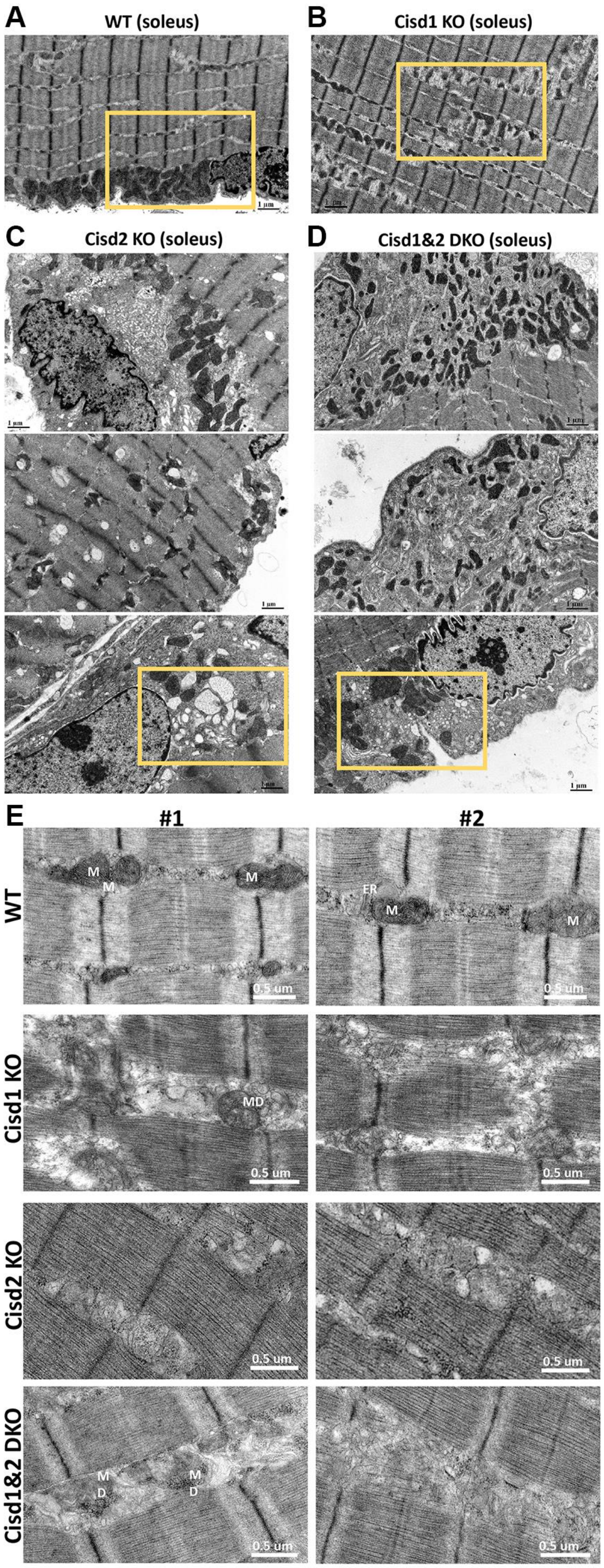

Supplementary Figure 3. Ultrastructure (low-powered) of skeletal muscle (soleus) in Cisd1&2 DKO mice. (**A**) Architecture of soleus in WT mice. (**B**) Mitochondrial defect, myofibril degeneration and ER stress in Cisd1 KO soleus. (**C**) Mitochondrial defect, myofibril degeneration, necrosis and ER stress in Cisd2 KO soleus. (**D**) Mitochondrial defect, myofibril degeneration, necrosis and ER stress in Cisd1&2 DKO soleus. (**E**) Interfibrillar mitochondrial and ER defects in Cisd1 KO, Cisd2 and Cisd1&2 DKO gastrocnemius. Abbreviations: M: mitochondria; MD: Mitochondrial defect. Mouse age, 5 weeks old.

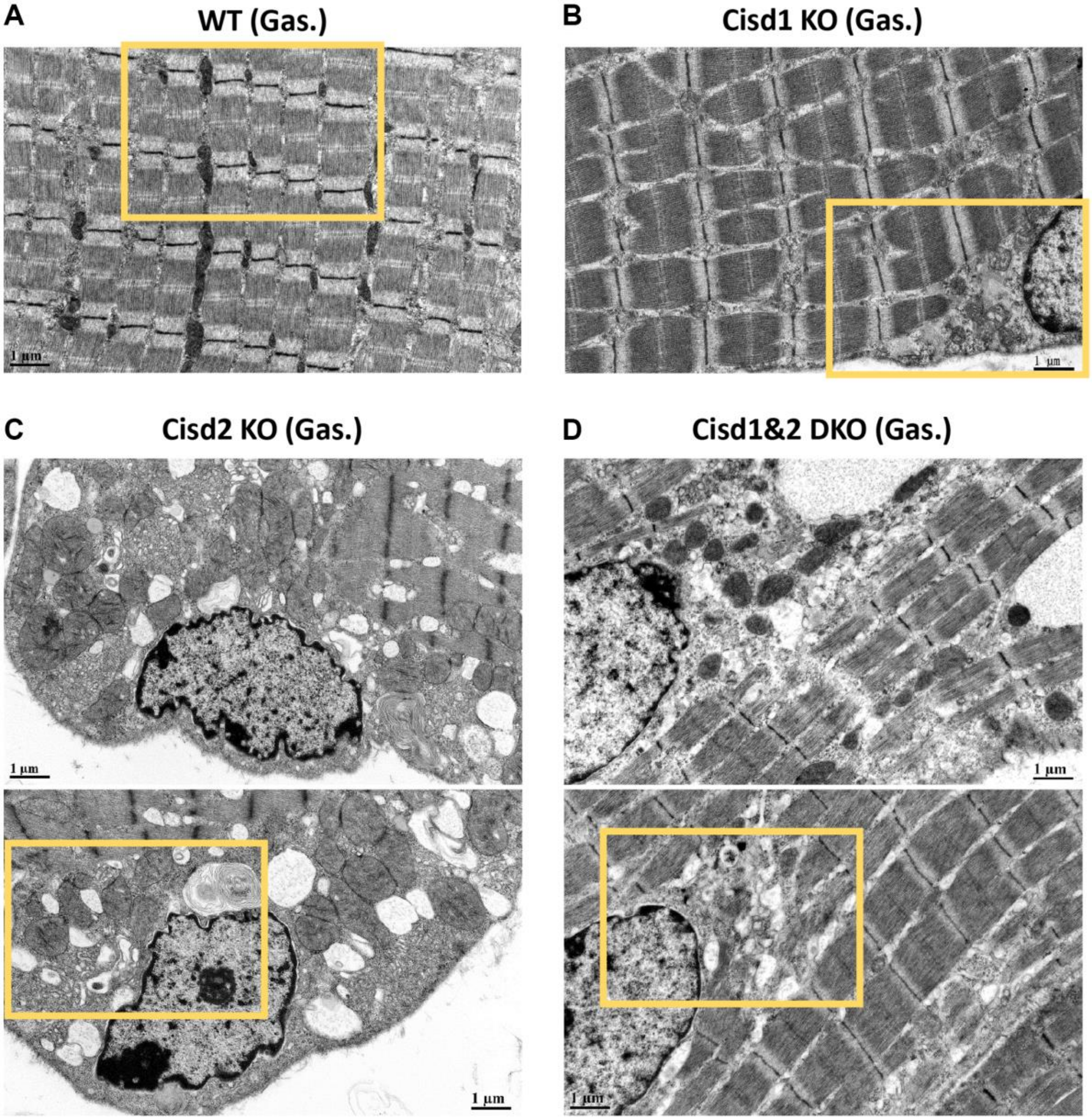

Supplementary Figure 4. Ultrastructure (low-powered) of skeletal muscle (Gastrocnemius, Gas.) in Cisd1&2 DKO mice. (**A**) Architecture of soleus in WT mice. (**B**) Mitochondrial defect, necrosis and ER stress in Cisd1 KO soleus. (**C**) Mitochondrial defect, necrosis and ER stress in Cisd2 KO soleus. (**D**) Mitochondrial defect, myofibril degeneration, necrosis and ER stress in Cisd1&2 DKO soleus. Mouse age, 5 weeks old.

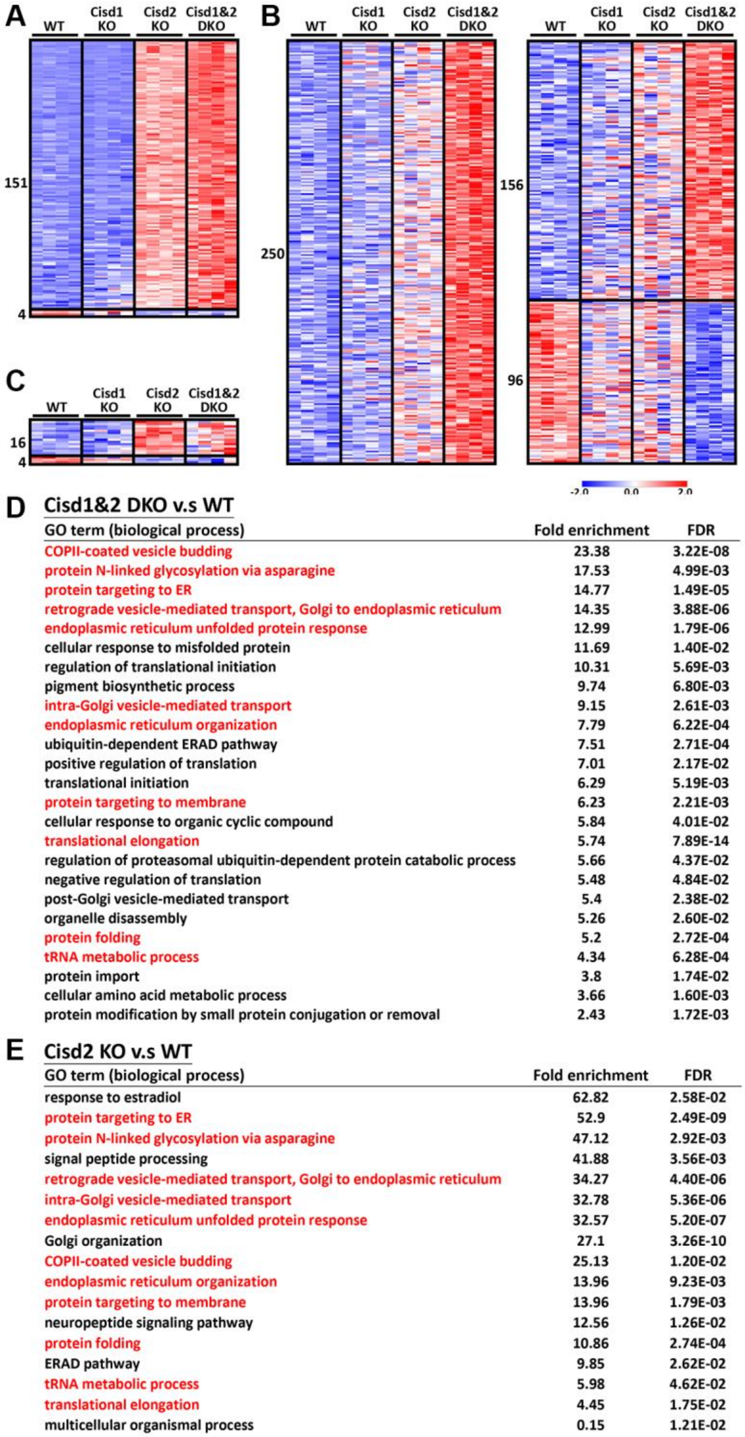

D **Cisd1&2 DKO v.s WT**

GO term (biological process)	Fold enrichment	FDR
COPII-coated vesicle budding	23.38	3.22E-08
protein N-linked glycosylation via asparagine	17.53	4.99E-03
protein targeting to ER	14.77	1.49E-05
retrograde vesicle-mediated transport, Golgi to endoplasmic reticulum	14.35	3.88E-06
endoplasmic reticulum unfolded protein response	12.99	1.79E-06
cellular response to misfolded protein	11.69	1.40E-02
regulation of translational initiation	10.31	5.69E-03
pigment biosynthetic process	9.74	6.80E-03
intra-Golgi vesicle-mediated transport	9.15	2.61E-03
endoplasmic reticulum organization	7.79	6.22E-04
ubiquitin-dependent ERAD pathway	7.51	2.71E-04
positive regulation of translation	7.01	2.17E-02
translational initiation	6.29	5.19E-03
protein targeting to membrane	6.23	2.21E-03
cellular response to organic cyclic compound	5.84	4.01E-02
translational elongation	5.74	7.89E-14
regulation of proteasomal ubiquitin-dependent protein catabolic process	5.66	4.37E-02
negative regulation of translation	5.48	4.84E-02
post-Golgi vesicle-mediated transport	5.4	2.38E-02
organelle disassembly	5.26	2.60E-02
protein folding	5.2	2.72E-04
tRNA metabolic process	4.34	6.28E-04
protein import	3.8	1.74E-02
cellular amino acid metabolic process	3.66	1.60E-03
protein modification by small protein conjugation or removal	2.43	1.72E-03

E **Cisd2 KO v.s WT**

GO term (biological process)	Fold enrichment	FDR
response to estradiol	62.82	2.58E-02
protein targeting to ER	52.9	2.49E-09
protein N-linked glycosylation via asparagine	47.12	2.92E-03
signal peptide processing	41.88	3.56E-03
retrograde vesicle-mediated transport, Golgi to endoplasmic reticulum	34.27	4.40E-06
intra-Golgi vesicle-mediated transport	32.78	5.36E-06
endoplasmic reticulum unfolded protein response	32.57	5.20E-07
Golgi organization	27.1	3.26E-10
COPII-coated vesicle budding	25.13	1.20E-02
endoplasmic reticulum organization	13.96	9.23E-03
protein targeting to membrane	13.96	1.79E-03
neuropeptide signaling pathway	12.56	1.26E-02
protein folding	10.86	2.74E-04
ERAD pathway	9.85	2.62E-02
tRNA metabolic process	5.98	4.62E-02
translational elongation	4.45	1.75E-02
multicellular organismal process	0.15	1.21E-02

Supplementary Figure 5. RNA sequencing analyses to examine the DEGs for the Cisd1&2 DKO, Cisd1 KO, Cisd2 KO and WT mice. (**A**) The heatmap of gene expression in both of DKO and Cisd2 KO muscles (155 genes). (**B**) The heatmap of gene expression in DKO muscles alone (502 genes). (**C**) The heatmap of gene expression in Cisd2 KO muscles alone (20 genes). (**D**) The Go enrichment analysis of RNA sequencing data in Cisd1&2 DKO muscles. (**E**) The Go enrichment analysis of RNA sequencing data in Cisd2 KO muscles. Highlight in red indicates the biological process had significant changes in both CIsd2KO and Cisd1&2 DKO muscles.
