# Supplementary material for: Cisd1 synergizes with Cisd2 to modulate protein processing by maintaining mitochondrial and ER homeostasis
Source: Aging (Albany NY). 2025 May 8;17(5):1275–97. doi: 10.18632/aging.206249 (PMC12151500; doi:10.18632/aging.206249)
Supplement: Supplementary Table 1 [file aging-17-206249-s002.pdf]

## SUPPLEMENTARY TABLE

**Supplementary Table 1. The calculation of the *p*-values for the body weight analysis of the different mouse groups.**

| Male/Age (week) | ALL | WT vs. Cisd1 KO | WT vs. Cisd2 KO | WT vs. DKO | Cisd1 KO vs. Cisd2 KO | Cisd1 KO vs. DKO | Cisd2 KO vs. DKO |
|---|---|---|---|---|---|---|---|
| | | | | A | | | |
| 2 | 0.0520 | >0.9999 | >0.9999 | >0.9999 | 0.0390 | 0.8255 | 0.8003 |
| 3 | 0.0010 | 0.7375 | >0.9999 | 0.7858 | 0.2305 | 0.0004 | >0.9999 |
| 4 | <0.0001 | >0.9999 | 0.7784 | 0.0020 | 0.3176 | <0.0001 | 0.3303 |
| 5 | <0.0001 | >0.9999 | 0.1079 | <0.0001 | 0.3872 | <0.0001 | 0.2667 |
| 6 | <0.0001 | >0.9999 | 0.0588 | <0.0001 | 0.1625 | <0.0001 | 0.6714 |
| 7 | <0.0001 | >0.9999 | 0.0085 | <0.0001 | 0.0143 | <0.0001 | >0.9999 |
| 8 | <0.0001 | >0.9999 | 0.0061 | <0.0001 | 0.0115 | <0.0001 | >0.9999 |
| 9 | <0.0001 | >0.9999 | 0.0083 | <0.0001 | 0.0113 | <0.0001 | >0.9999 |
| 10 | <0.0001 | >0.9999 | 0.0087 | <0.0001 | 0.0104 | <0.0001 | >0.9999 |
| 11 | <0.0001 | >0.9999 | 0.0061 | <0.0001 | 0.0031 | <0.0001 | >0.9999 |
| 12 | <0.0001 | >0.9999 | 0.0038 | <0.0001 | 0.0036 | <0.0001 | >0.9999 |
| 13 | <0.0001 | >0.9999 | 0.0039 | <0.0001 | 0.0073 | <0.0001 | >0.9999 |
| 14 | <0.0001 | >0.9999 | 0.0058 | <0.0001 | 0.0061 | <0.0001 | >0.9999 |
| 15 | <0.0001 | >0.9999 | 0.0029 | <0.0001 | 0.0034 | <0.0001 | >0.9999 |
| 16 | <0.0001 | >0.9999 | 0.0026 | <0.0001 | 0.0027 | <0.0001 | >0.9999 |
| 17 | <0.0001 | >0.9999 | 0.0033 | <0.0001 | 0.0023 | <0.0001 | >0.9999 |
| 18 | <0.0001 | >0.9999 | 0.0016 | <0.0001 | 0.0022 | <0.0001 | >0.9999 |
| 19 | <0.0001 | >0.9999 | 0.0017 | <0.0001 | 0.0028 | <0.0001 | >0.9999 |
| 20 | <0.0001 | >0.9999 | 0.0019 | <0.0001 | 0.0025 | <0.0001 | >0.9999 |
| 21 | <0.0001 | >0.9999 | 0.0038 | <0.0001 | 0.0022 | <0.0001 | >0.9999 |
| 22 | <0.0001 | >0.9999 | 0.0008 | <0.0001 | 0.0015 | <0.0001 | >0.9999 |
| 23 | <0.0001 | >0.9999 | 0.0007 | <0.0001 | 0.0010 | <0.0001 | >0.9999 |
| 24 | <0.0001 | >0.9999 | 0.0011 | <0.0001 | 0.0008 | <0.0001 | >0.9999 |
| | | | | B | | | |
| 2 | 0.3519 | >0.9999 | >0.9999 | 0.6170 | >0.9999 | 0.9887 | >0.9999 |
| 3 | 0.0305 | >0.9999 | >0.9999 | 0.0512 | >0.9999 | 0.0368 | >0.9999 |
| 4 | <0.0001 | 0.1304 | 0.0244 | <0.0001 | >0.9999 | 0.0053 | >0.9999 |
| 5 | <0.0001 | 0.0229 | 0.0176 | <0.0001 | >0.9999 | 0.0074 | 0.8793 |
| 6 | <0.0001 | 0.0076 | 0.0061 | <0.0001 | >0.9999 | 0.0300 | >0.9999 |
| 7 | <0.0001 | 0.0151 | 0.0010 | <0.0001 | 0.5811 | 0.0046 | >0.9999 |
| 8 | <0.0001 | 0.0342 | 0.0014 | <0.0001 | 0.4466 | 0.0010 | >0.9999 |
| 9 | <0.0001 | 0.0181 | 0.0017 | <0.0001 | 0.6049 | 0.0013 | >0.9999 |
| 10 | <0.0001 | 0.0248 | 0.0005 | <0.0001 | 0.2907 | 0.0007 | >0.9999 |
| 11 | <0.0001 | 0.0205 | 0.0014 | <0.0001 | 0.4972 | 0.0004 | >0.9999 |
| 12 | <0.0001 | 0.0170 | 0.0012 | <0.0001 | 0.5015 | 0.0007 | >0.9999 |
| 13 | <0.0001 | 0.0191 | 0.0321 | <0.0001 | >0.9999 | 0.0003 | >0.9999 |
| 14 | <0.0001 | 0.0147 | 0.0219 | <0.0001 | >0.9999 | 0.0004 | >0.9999 |
| 15 | <0.0001 | 0.0160 | 0.0508 | <0.0001 | >0.9999 | 0.0011 | >0.9999 |
| 16 | <0.0001 | 0.0118 | 0.0367 | <0.0001 | >0.9999 | 0.0010 | >0.9999 |
| 17 | <0.0001 | 0.0213 | 0.0008 | <0.0001 | 0.2737 | 0.0007 | >0.9999 |
| 18 | <0.0001 | 0.0219 | 0.0002 | <0.0001 | 0.1539 | 0.0006 | >0.9999 |
| 19 | <0.0001 | 0.0346 | 0.0004 | <0.0001 | 0.1931 | 0.0003 | >0.9999 |

| | | | | | | | |
|---|---|---|---|---|---|---|---|
| 20 | <0.0001 | 0.0355 | 0.0004 | <0.0001 | 0.1933 | 0.0001 | >0.9999 |
| 21 | <0.0001 | 0.0506 | 0.0002 | <0.0001 | 0.1008 | 0.0002 | >0.9999 |
| 22 | <0.0001 | 0.0327 | 0.0003 | <0.0001 | 0.1511 | 0.0002 | >0.9999 |
| 23 | <0.0001 | 0.0284 | 0.0002 | <0.0001 | 0.1459 | 0.0002 | >0.9999 |
| 24 | <0.0001 | 0.0522 | 0.0002 | <0.0001 | 0.0948 | 0.0001 | >0.9999 |

*Yellow highlight indicates $p < 0.05$; pink highlight indicates $p < 0.005$. For growth curve analysis, the Kruskal-Wallis test with Dunn's multiple comparisons test was used to determine the statistical significance at each time point. (A) Male. (B) Female.
